# Kinesiophobia following ACL reconstruction: a qualitative netnographic study of lived experiences shared on Reddit

**DOI:** 10.3389/fpsyg.2026.1926382

**Published:** 2026-10-14

**Authors:** Yakup Kahve, Yagmur Darben Azarsiz, Serap Aydin, Tahsin Aydin, Utku Gurhan, Aybeniz Civan Kahve

**Affiliations:** 1 Department of Orthopaedics and Traumatology, Ankara Bilkent City Hospital, Ankara, Türkiye; 2 Department of Psychiatry, Baris Mental and Neurological Diseases Hospital, Nicosia, Cyprus; 3 Department of Psychiatry, Ankara Bilkent City Hospital, Ankara, Türkiye; 4 Department of Orthopaedics and Traumatology, Faculty of Medicine, Kyrenia University, Kyrenia, Cyprus; 5 Department of Psychiatry, Faculty of Medicine, Gazi University, Ankara, Türkiye

**Keywords:** anterior cruciate ligament reconstruction, biopsychosocial, fear of movement, kinesiophobia, netnography, qualitative research, rehabilitation psychology

## Abstract

**Introduction:**

Kinesiophobia, broadly defined as fear of movement and vulnerability to (re)injury, is one of the most significant psychological barriers to functional recovery following anterior cruciate ligament (ACL) reconstruction, yet its everyday, lived texture is not fully captured by structured psychometric instruments. Orthopedic clinicians frequently observe that patients who have achieved objective surgical and functional recovery nonetheless remain reluctant to resume movement. This study aimed to explore this phenomenon in patients’ own words.

**Methods:**

We conducted a qualitative netnographic study of 50 first-person Reddit narratives shared by individuals with ACL injury, spanning the rehabilitation trajectory from preoperative anxiety to chronic post-return instability. Data were analyzed using Braun and Clarke’s reflexive thematic analysis by an interdisciplinary team of orthopedic surgeons and psychiatrists.

**Results:**

Three interconnected themes emerged. First, a progressive loss of trust in the injured body, in which normal postoperative sensations were hypervigilantly monitored and catastrophically reinterpreted as signs of graft failure, persisting even after objective strength testing and medical clearance. Second, fear-driven disruption of functional identity and future planning, in which reinjury fear extended beyond the knee to threaten occupational continuity, financial security, and physical self-concept. Third, persistent uncertainty and compulsive reassurance-seeking, fueled by inconsistent rehabilitation guidance and unfavorable social comparison with peers’ recovery timelines on social media.

**Discussion:**

Psychological readiness for return to activity appears to be shaped by an interacting network of bodily, identity-related, and informational factors that extend beyond fear of reinjury as traditionally measured. Incorporating expectation management, structured reassurance, and psychologically informed communication into postoperative care protocols may benefit orthopedic and rehabilitation teams. Naturally occurring online narratives may serve as an ecologically valid source of insight into the psychological dimensions of ACL recovery, complementing structured psychometric assessment.

## 1 Introduction

### 1.1 Background: ACL injury and recovery outcomes

Anterior cruciate ligament (ACL) injuries are among the most common knee injuries, particularly in young and physically active populations, and their surgical and rehabilitation management represents a major focus within sports medicine (Grindem et al., 2016). Although technological advances in modern orthopedic surgery and increasingly refined graft techniques have achieved high rates of mechanical stability, return-to-sport and return-to-function outcomes have not yet reached the desired levels (Ardern et al., 2012). Current literature suggests that anatomically successful surgical outcomes do not always progress in parallel with psychological wellbeing and subjective perceptions of functional confidence (Christino et al., 2016; Carter et al., 2020).

### 1.2 The psychological dimension: kinesiophobia after ACL injury

One of the most important psychological factors affecting rehabilitation following ACL injury is kinesiophobia, most commonly defined as an excessive, often irrational fear of movement and vulnerability to (re)injury; in the postoperative context, this fear typically develops in response to the prior injury itself and the perceived physical fragility of the healing joint (Bullock et al., 2022). Individuals experiencing kinesiophobia may avoid movement because of concerns about damaging the knee joint or sustaining another injury, which has been associated with neuromuscular inhibition, reduced physical performance, and prolonged rehabilitation processes (Bullock et al., 2022; Clifford et al., 2017). Particularly during return-to-sport phases, persistent feelings of insecurity may affect not only physical capacity but also self-efficacy and social identity (Flosadottir et al., 2018; Bullock et al., 2022).

Recent studies suggest that fear experienced after ACL injury is not solely an individual psychological reaction, but rather a multidimensional phenomenon shaped by economic concerns, social expectations, performance pressure, and healthcare-related experiences (Gamble et al., 2024). Uncertainty during rehabilitation, hypervigilance toward knee-related sensations such as clicking or instability, and exposure to conflicting health information from multiple sources may negatively influence rehabilitation adherence (Saxena et al., 2020; Gamble et al., 2024). In addition, contextual life factors such as fear of job loss, financial pressure, or concerns regarding athletic career continuity may also shape rehabilitation behaviors (Secrist et al., 2016; Rosenstiel et al., 2019).

### 1.3 Clinical observation and the present knowledge gap

The impetus for this study arose from a recurrent clinical observation among the orthopedic members of our research team. A substantial subset of patients who had achieved satisfactory graft healing, symmetric strength, and unremarkable clinical and radiological findings after ACL reconstruction nonetheless remained reluctant to resume movement, sport, or full daily activity. This discrepancy between objective surgical recovery and persistent behavioral avoidance prompted an interdisciplinary discussion with psychiatric colleagues, who identified the pattern as consistent with kinesiophobia. Postoperative rehabilitation protocols routinely address the biomechanical and functional aspects of return to activity, yet they rarely incorporate a structured mental health component, despite the clinical relevance of fear-related cognitions to recovery outcomes.

What remains poorly understood is how this fear is actually lived. Existing knowledge of kinesiophobia rests largely on structured questionnaires, clinical examinations, and controlled follow-up protocols, which quantify how much fear a patient reports but say little about how that fear is experienced, interpreted, and narrated in everyday life. Because fear-avoidance is highly contextual, recent research has suggested that it may be understood most fully through qualitative approaches that preserve patients’ own language and the situations in which their concerns arise. With the growing prevalence of digital health communities, social media platforms have emerged as one such source, allowing individuals to share health-related experiences in natural and relatively unfiltered ways. Reddit, in particular, combines anonymity with a discussion format built around first-person, narrative-style posts rather than short structured comments, which allows users to describe pain, fear, rehabilitation experiences, and healthcare interactions in extended, spontaneous prose. This combination of anonymity and narrative format makes Reddit a well-suited, though not the only possible, data source for capturing the lived experience of kinesiophobia.

In recent years, the use of social media data in health research has increased substantially, and online patient narratives have been shown to provide valuable insights that complement traditional clinical assessments (Farsi et al., 2022; Drewniak et al., 2020). One approach with particular promise for this kind of material is netnography, a qualitative methodology developed for the study of naturally occurring narratives within online communities. Netnography allows patient experience to be examined in the setting where it is spontaneously expressed, rather than in the setting of a clinical interview, and it is increasingly used in health research for precisely this reason. Experiences shared on anonymous online platforms may thereby contribute to a deeper understanding of reinjury fear, rehabilitation motivation, and perceptions toward healthcare professionals.

Accordingly, we examined first-person Reddit posts shared by individuals who had experienced an ACL injury. The sample was not limited to competitive or recreational athletes, but encompassed anyone describing fear-related experiences during ACL rehabilitation. Our research question was: how do individuals recovering from ACL injury describe and make sense of movement-related fear, and what somatic, psychological, social, and clinical dimensions characterize this experience in their own words? In addressing this question, we sought to contribute to a fuller understanding of patient experience during ACL rehabilitation. We also sought to provide an evidence-informed rationale for integrating a structured mental health component into orthopedic rehabilitation practice, alongside existing biomechanical and functional protocols.

## 2 Methods

### 2.1 Study design

This study was designed as a qualitative netnographic investigation exploring fear-related experiences and kinesiophobia narratives among individuals reporting anterior cruciate ligament (ACL) injuries on Reddit. A reflexive thematic analysis (RTA) framework (Braun and Clarke, 2006) was employed to examine naturally occurring patient narratives shared in online communities. Data were interpreted through a biopsychosocial perspective, i.e., a conceptual framework in which recovery is understood as the product of interacting biological (graft healing, strength, mechanical stability), psychological (fear, hypervigilance, catastrophic interpretation), and social (occupational, financial, and relational) factors, rather than biomechanical status alone; this perspective guided how psychological distress, perceived instability, rehabilitation experiences, and social concerns were interpreted in user-generated content. Reporting of the study was informed by the Standards for Reporting Qualitative Research (O’Brien et al., 2014).

Netnography and RTA served distinct but complementary functions in this design. Netnography is the overarching methodological approach used to identify, ethically access, and interpret naturally occurring data within an online community, and it governed platform selection, immersion in the community context, and data-handling procedures. RTA is the analytic method applied once the narrative data had been assembled, and it was used to identify and interpret patterns of meaning across the collected posts. Together, the two allowed patient experience to be examined within its natural digital environment and then interpreted systematically at the level of meaning. RTA was selected as the analytic method because it suited both the expertise of the research team and our aim of producing clinically relevant interpretation. RTA foregrounds the active, reflexive role of the researcher in generating themes, which mattered here because our six-member team combined orthopedic and psychiatric perspectives. It offered a structured way to make those disciplinary assumptions visible and to interrogate them throughout coding, rather than treating them as bias to be eliminated. RTA is also well suited to identifying patterns of meaning shared across a set of separate, relatively short first-person accounts, and it preserves the contextual and emotional texture of those accounts.

### 2.2 Data source and search strategy

Data were collected from Reddit (Reddit Inc., San Francisco, CA, USA), a publicly accessible social media platform characterized by anonymous and community-based discussion structures. As outlined in the Introduction, Reddit was selected in preference to more tightly moderated or identity-linked platforms because its combination of anonymity and a long-form, first-person posting format (as opposed to short, reactive comments) allows users to share detailed health-related experiences in a relatively spontaneous and less socially constrained way, with fewer of the reputational or professional-identity concerns that can constrain disclosure on platforms such as Facebook or Instagram.

The search process was conducted manually using Reddit’s publicly accessible internal search engine (https://www.reddit.com/search/). Searches were performed on April 25, 2026, using the keywords “ACL” and “kinesiophobia” entered as combined search terms. The clinical term “kinesiophobia” was used, rather than lay expressions such as “scared to run” or “afraid to reinjure,” because pilot browsing of r/ACL indicated that this term itself was already used by a substantial number of posters, likely reflecting its increasing lay familiarity through orthopedic patient education materials and peer discussion; combining it with “ACL” was intended to prioritize posts with explicit, self-recognized fear-avoidance content. We acknowledge that this keyword choice excluded narratives that described comparable fear-related experiences using only lay terminology, which is addressed as a limitation. Reddit’s default relevance-based ranking system was used together with the “all time” temporal filter in order to identify posts across the platform’s available historical archive.

Posts were screened sequentially according to the order generated by Reddit’s relevance-based search algorithm. Most included narratives originated from r/ACL, a large publicly accessible Reddit community focused on ACL injury experiences and rehabilitation discussions, although posts from other publicly accessible subreddits appearing within the search results were also evaluated when relevant to the study aims.

Because Reddit’s search interface uses a dynamically generated and proprietary relevance-ranking system together with an infinite-scroll display structure, the exact ordering and total number of retrievable posts may vary across users and time points. Accordingly, the present study did not aim to establish an exhaustive census of all ACL-related Reddit content, but rather to obtain conceptually rich experiential narratives relevant to the study aims.

Only original written posts created by users were included in the analysis. Comment sections, replies, reposts, and non-textual submissions were excluded in order to preserve the integrity of individual narratives and minimize interpretive noise during thematic coding.

### 2.3 Sampling and eligibility criteria

A purposive sampling strategy was used to identify posts containing detailed experiential narratives related to ACL injury and movement-related fear. Posts were eligible for inclusion if they:

1. Included explicit references to clinical evaluation processes such as physician consultation, magnetic resonance imaging (MRI), orthopedic assessment, surgery, or rehabilitation,
2. Described psychological, functional, or emotional experiences related to movement avoidance, reinjury fear, instability perception, or rehabilitation anxiety,
3. Contained sufficiently detailed first-person narratives relevant to the study aims (e.g., a multi-sentence post describing a specific fear-related episode, such as freezing during activity after hearing a knee click, rather than a single unelaborated sentence such as “I’m scared to run”).

Posts were excluded if they:

1. Consisted primarily of video content without meaningful written narrative,
2. Included only brief, nonspecific, or non-experiential expressions lacking clinical or psychological relevance,
3. Were unrelated to ACL injury or kinesiophobia-related experiences,
4. Represented duplicate or substantially repetitive content identified during later stages of analysis.

A total of 137 original Reddit posts were screened during the review process; throughout this manuscript, we use “post” to refer to the raw Reddit submission as it appeared on the platform, and “narrative” to refer to the same content once it had been judged eligible, extracted, and treated as a unit of analysis. Every included narrative therefore originated from a post, but not every screened post met the criteria to become an analyzed narrative. During eligibility assessment, 8 predominantly video-based submissions, 7 irrelevant or non-narrative posts, 54 brief or insufficiently experiential posts, and 18 duplicate or substantially repetitive posts were excluded. Sampling and analysis proceeded concurrently and iteratively until thematic saturation was achieved, defined as the point at which additional narratives no longer meaningfully expanded the conceptual scope, thematic structure, or interpretive understanding of the dataset. At later stages of coding, incoming narratives largely reinforced existing thematic categories without generating substantively new codes or interpretive dimensions, supporting the determination that thematic saturation had been achieved. A flow diagram summarizing the screening and narrative selection process is presented in Figure 1. Representative quotations were selected to illustrate recurrent thematic patterns while minimizing identifiability and preserving narrative meaning.

**Figure 1:**
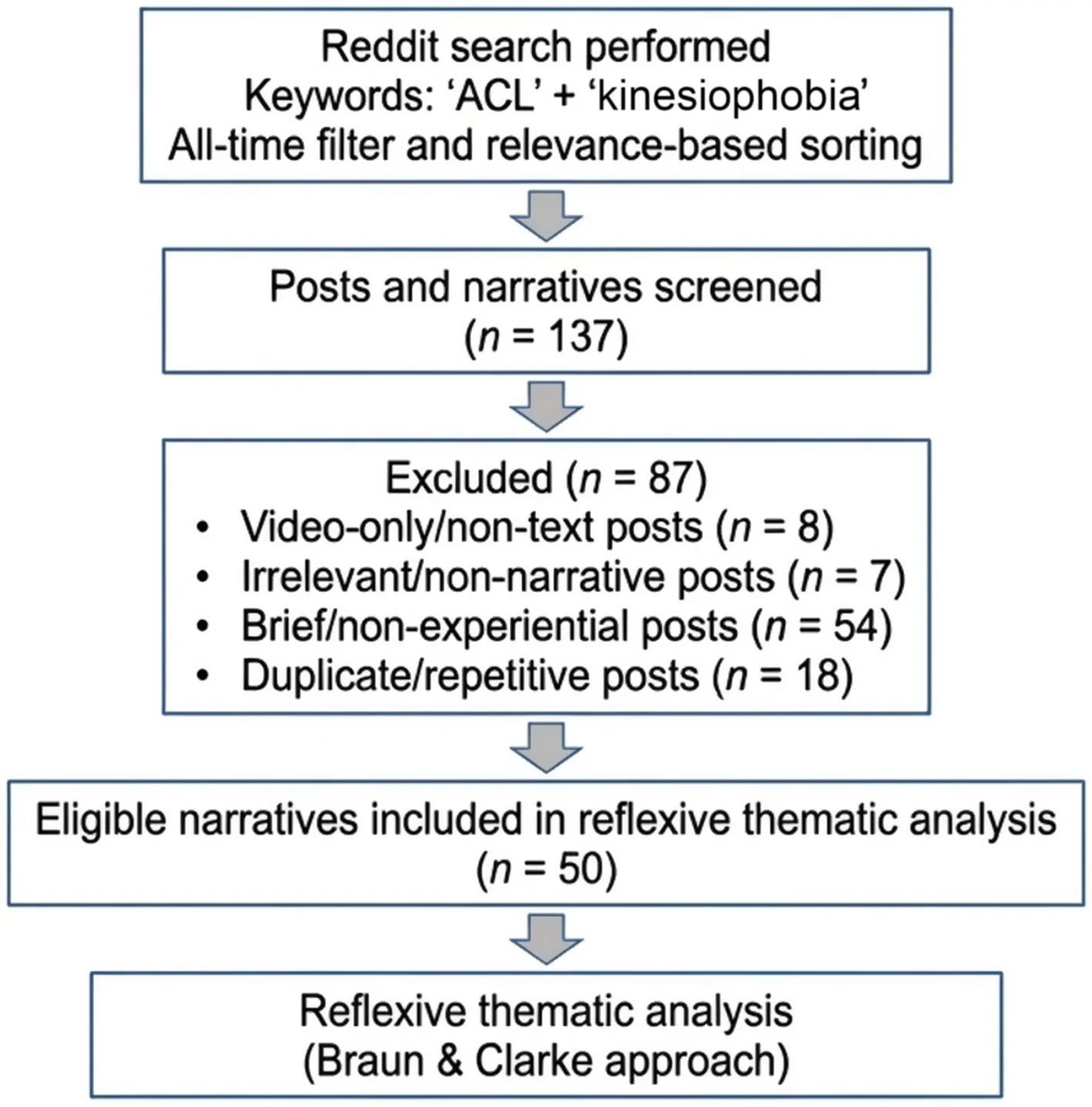
Flow diagram of Reddit narrative screening, eligibility assessment, and inclusion process used in the present qualitative study. Narratives were sequentially screened using purposive sampling until thematic saturation was achieved; ACL, anterior cruciate ligament.

### 2.4 Data extraction and management

Eligible posts were manually transferred into a structured Microsoft Word document by six researchers, comprising three orthopedic surgeons and three psychiatrists, who were responsible for screening, eligibility assessment, and initial narrative review. All original posts were already in English; to minimize errors introduced during this manual copy-paste transfer (e.g., accidental truncation or duplication of text) and to preserve contextual authenticity, the original English-language wording was copied verbatim into the coding document for internal analytic purposes only. No translation was involved. Each included narrative corresponded to one complete Reddit post and was assigned an anonymized identifier (e.g., Narrative 1, Narrative 2); narrative length varied considerably across the dataset, ranging from short focused accounts of a single fear-related episode to longer posts describing the full rehabilitation trajectory. All potentially identifying information, including usernames, dates, and location references, was removed prior to analysis. For publication, all illustrative excerpts presented in the Results section were subsequently paraphrased (see Section 2.6) to minimize the risk of re-identification via reverse text-search.

### 2.5 Data analysis

Data were analyzed using Braun and Clarke’s six-phase reflexive thematic analysis approach. An inductive coding strategy was adopted to allow themes to emerge directly from narratives rather than imposing predetermined conceptual categories. Coding was performed manually using structured Word and Excel tables rather than dedicated qualitative data analysis software; each narrative was entered as a separate row, with emerging codes, illustrative excerpts, and analytic memos recorded in adjacent columns to preserve transparency and traceability throughout the coding process. A structured codebook summarizing the final coding framework, code definitions, and illustrative (paraphrased) excerpts is provided in Supplementary Table S1.

Initially, all researchers independently reviewed the dataset multiple times to achieve familiarization with the content. Meaning units and recurring experiential patterns were then coded systematically. Following initial coding, codes were iteratively compared, refined, and grouped into broader thematic categories through consensus-based discussions among the research team.

Analytic rigor was strengthened through investigator triangulation, interdisciplinary interpretation, and repeated comparison between coded extracts and the original narratives. Disagreements regarding coding or thematic categorization were resolved through discussion until consensus was achieved.

Researcher reflexivity was considered throughout the analytic process. The interdisciplinary backgrounds of the investigators, comprising orthopedic surgeons and psychiatrists, informed interpretation of the narratives while also requiring ongoing reflexive discussion regarding potential disciplinary assumptions and interpretive bias during coding and thematic development.

### 2.6 Ethical considerations

This study utilized publicly available online data obtained from an open-access digital platform without direct interaction with users. In accordance with prevailing ethical guidelines for internet-mediated research and the principles of the Declaration of Helsinki, the study was considered an analysis of publicly accessible anonymous data.

Because the study analyzed publicly accessible anonymous online narratives without direct participant interaction or identifiable personal data, formal institutional review board approval was not required according to local institutional policies.

To protect user privacy, all identifiable information and metadata were removed during data extraction. No attempts were made to contact users, access private profiles, or perform digital tracking beyond the publicly visible content directly relevant to the research question. To reduce the risk of re-identification through reverse text-search, all narrative excerpts presented in this manuscript were paraphrased rather than reproduced verbatim, consistent with established netnographic data-masking practices, while the semantic and emotional content of each excerpt was preserved.

## 3 Results

A total of 50 Reddit narratives describing experiences related to ACL injury and kinesiophobia were included in the final analysis. The included narratives represented a broad spectrum of rehabilitation stages, including pre-operative, early post-operative, mid-stage rehabilitation, return-to-sport, revision surgery, and chronic instability experiences. Across narratives, ACL injury was consistently described not merely as a biomechanical impairment, but as a multidimensional disruption affecting bodily trust, psychological stability, occupational functioning, and future identity. Characteristics of the included narratives are summarized in Table 1.

**Table 1:** Sample and narrative characteristics of the reflexive thematic analysis dataset.

Variable	Description
Final included narratives	50
Primary data source	Public Reddit narratives
Primary subreddit represented	r/ACL^*^
Rehabilitation stages represented	Pre-operative, early post-operative, mid-rehabilitation, return-to-sport, revision surgery, chronic instability
Dominant narrative characteristics	Reinjury fear, bodily distrust, uncertainty, reassurance-seeking, psychosocial disruption
Analytic approach	Reflexive thematic analysis
Research team composition	Three orthopedic surgeons and three psychiatrists
Saturation approach	Iterative thematic saturation

^*^ACL, anterior cruciate ligament.

Consistent with the reflexive orientation of the analysis, theme development was shaped by ongoing discussion of the research team’s own disciplinary lenses. Orthopedic team members initially read several narratives primarily as accounts of insufficient rehabilitation guidance, whereas psychiatric team members were more attentive to catastrophic cognitive interpretation; reconciling these readings through iterative discussion, rather than resolving them into a single “correct” code, directly shaped the final structure and naming of the three themes presented below.

Reflexive thematic analysis identified three overarching themes:

1. Loss of Trust in the Injured Body
2. Fear-Driven Identity and Future Disruption
3. Uncertainty, Information Seeking, and the Need for Clinical Reassurance

### 3.1 Loss of trust in the injured body

The most prominent theme across narratives was the progressive loss of confidence in the injured body. Across narratives, ordinary postoperative sensations such as clicking, stiffness, instability, muscular inhibition, and transient pain were frequently interpreted as indicators of graft failure or irreversible structural damage. Many narratives reflected persistent hypervigilance toward bodily sensations and catastrophic interpretation of otherwise expected recovery-related experiences.

Across multiple narratives, normal joint sounds or minor movements were associated with intense anxiety and fear of reinjury:

“Any small pop or click in my knee makes my stomach lurch, and my first thought is always that I’ve retorn the graft.” (Narrative 7).

“I lost hours last night searching online to find out whether a graft can tear without any swelling.” (Narrative 30).

Others described profound feelings of bodily disconnection and altered proprioceptive awareness:

“My quad feels totally lifeless, as if the signal between my brain and that muscle has been switched off.” (Narrative 6).

“My sense of where my leg is in space feels completely broken. I move like an awkward stranger inside my own body.” (Narrative 36).

Despite satisfactory physical recovery, many narratives reflected persistent protective behaviors and movement-related caution:

“My strength tests show my operated leg matching the healthy one, but mentally I still handle it as if it might shatter.” (Narrative 23).

Fear of reinjury frequently persisted even after medical clearance for sports participation:

“During a basketball game, another player’s foot came close to mine and I froze on the spot.” (Narrative 4).

Collectively, these narratives reflected a discrepancy between objective physical recovery and subjective perceptions of bodily safety and control. This pattern directly echoed the clinical observation that motivated the study, in which orthopedic clearance for activity did not reliably translate into a patient’s own sense of readiness to move.

### 3.2 Fear-driven identity and future disruption

Beyond physical recovery concerns, narratives in this theme shared a common underlying structure: loss of confidence in the body’s reliability was described as threatening not only future movement, but also the specific life roles that depended on that body. These included paid work, caregiving, independent living, and, for some, sport or exercise as a valued activity rather than a career. We grouped occupational/functional identity and financial security within a single theme, rather than as separate themes, because narratives rarely described these concerns in isolation; instead, participants moved fluidly between them within the same passage, for example describing an inability to work as simultaneously a loss of income and a loss of self-concept. Fear narratives therefore extended beyond reinjury itself and frequently centered on uncertainty regarding occupational continuity, one’s sense of physical capability and identity more broadly (which for some participants was specifically athletic, and for others centered on manual work, parenting, or everyday independence), and dependence on others.

One narrative described the injury as an existential disruption affecting both career and self-concept:

“This single injury feels like it has wiped out my career, my sense of who I am, and my financial security all at once.” (Narrative 16).

Financial anxiety emerged repeatedly, particularly among individuals whose employment depended on physical performance:

“What frightens me most about needing surgery is not knowing how I would keep up with my mortgage payments.” (Narrative 50).

“I’m already losing income, and I keep worrying about how I’ll cover my mortgage.” (Narrative 50).

Narratives also reflected fear of dependency and loss of autonomy during recovery:

“I’m genuinely frightened about how I’ll get through the first couple of weeks after surgery.” (Narrative 5).

“Honestly, the idea of relying on someone else scares me more than I expected.” (Narrative 46).

Several narratives reflected long-term behavioral adaptation driven by persistent fear of recurrence:

“Since my second tear, I’ve become extremely careful and avoid anything that feels remotely risky.” (Narrative 48).

Narratives involving recurrent injuries frequently reflected feelings of hopelessness, emotional exhaustion, and chronic vulnerability:

“I still cannot wrap my head around having to go through this whole ordeal again from square one.” (Narrative 32).

Overall, ACL injury was frequently experienced not only as a temporary orthopedic condition, but also as a destabilizing life event with long-term psychosocial implications.

Cyclical biopsychosocial framework of persistent kinesiophobia following ACL injury is illustrated in Figure 2.

**Figure 2:**
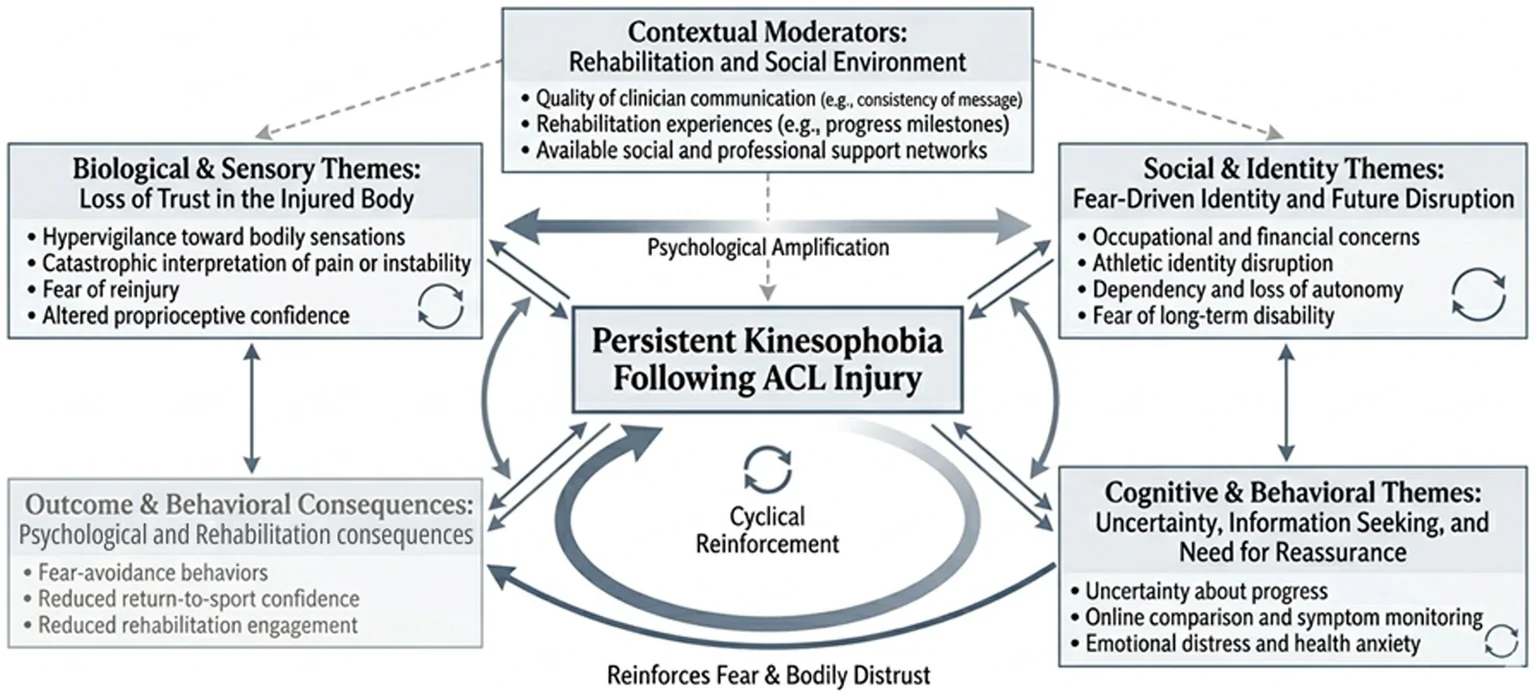
Cyclical biopsychosocial framework of persistent kinesiophobia following ACL injury. The figure illustrates the cyclical interactions between bodily distrust, identity-related disruption, uncertainty, reassurance-seeking behaviors, and rehabilitation experiences following ACL injury. Contextual factors may influence the persistence of fear-related cognitions during rehabilitation; ACL, anterior cruciate ligament.

### 3.3 Uncertainty, information seeking, and the need for clinical reassurance

A third major theme involved uncertainty surrounding rehabilitation, lack of clear clinical guidance, and compulsive information-seeking behaviors. Narratives frequently reflected reliance on internet-based information, social media comparisons, and repeated reassurance-seeking to manage anxiety regarding recovery progression.

Preoperative uncertainty and anticipatory anxiety were particularly common:

“I keep coming across frightening stories online about permanent kneeling pain and grafts failing.” (Narrative 2).

“I am absolutely terrified.” (Narrative 44).

Several narratives reflected distress associated with inadequate rehabilitation guidance or inconsistent clinical communication:

“I still have not received a physical therapy schedule and feel like I have no direction at all.” (Narrative 37).

“I feel frustrated and hemmed in by the rehabilitation protocol I’ve been given.” (Narrative 34).

Narratives also frequently described comparisons between personal recovery trajectories and those of others online, which appeared to intensify feelings of inadequacy and frustration:

“Watching other people online already running again by three months makes me feel like I’m falling behind.” (Narrative 11).

Internet searching and reassurance-seeking behaviors emerged repeatedly throughout the dataset:

“For anyone who has retorn their ACL, what symptoms did you notice beforehand?” (Narrative 41).

“What does knee instability actually feel like for those who have it?” (Narrative 42).

Collectively, these narratives suggested that uncertainty and inconsistent reassurance may contribute substantially to the persistence of fear-related cognitions during ACL rehabilitation.

Representative themes and quotations are summarized in Table 2.

**Table 2:** Overarching themes, thematic descriptions, and representative quotations.

Theme	Core thematic meaning	Representative quotation	Clinical/interpretive relevance
Loss of Trust in the Injured Body	Persistent hypervigilance, altered bodily confidence, and catastrophic interpretation of postoperative sensations	*“I still handle my leg as if it might shatter.”* (Narrative 23)	Suggests discrepancy between objective physical recovery and perceived bodily safety during rehabilitation
Fear-Driven Identity and Future Disruption	ACL^*^ injury experienced as a threat to occupational identity, independence, financial stability, and future functioning	*“This injury feels like it has wiped out my career, my identity, and my financial security.”* (Narrative 16)	Highlights the broader psychosocial consequences of ACL rehabilitation beyond reinjury fear
Uncertainty, Information Seeking, and Need for Clinical Reassurance	Anxiety reinforced by uncertainty, online comparison behaviors, and inconsistent rehabilitation guidance	*“I still have not received a physical therapy schedule and feel like I have no direction at all.”* (Narrative 37)	Suggests the importance of clinician communication and reassurance during rehabilitation

^*^ACL, anterior cruciate ligament.

## 4 Discussion

We asked how individuals recovering from ACL injury describe and make sense of movement-related fear in their own words. Analysis of narratives shared on Reddit by individuals experiencing ACL injury-related kinesiophobia identified three interconnected themes: loss of trust in the injured body, fear-driven identity and future disruption, and uncertainty-related reassurance seeking. It is already well established that recovery following ACL injury extends beyond biomechanical healing alone; the contribution of the present findings lies in specifying, in patients’ own language, which subjective experiences constitute that broader recovery process and how they interrelate. While contemporary ACL reconstruction techniques demonstrate high rates of mechanical stabilization, the narratives analyzed in this study indicate that subjective perceptions of safety, bodily reliability, and future functioning may remain substantially impaired despite clinical recovery (Ardern et al., 2012; Bullock et al., 2022).

### 4.1 Loss of bodily trust and catastrophic interpretation of sensations

One of the most prominent findings of this study was the persistent mistrust directed toward the injured body. Across narratives, common postoperative sensations such as clicking, stiffness, transient instability, or muscular inhibition were frequently interpreted as signs of graft failure or irreversible structural damage. These findings are consistent with previous literature describing fear-avoidance behaviors and hypervigilance following ACL reconstruction (Hoch et al., 2019; Ricketts, 2025).

Importantly, the narratives analyzed in this study suggest that patients may assign greater emotional significance to subjective bodily sensations than to formal clinical reassurance. Even among individuals who had achieved satisfactory physical recovery or clearance for return to sport, fear-related cognitions frequently persisted. This discrepancy between objective recovery markers and perceived bodily safety may help explain why successful surgical outcomes do not always translate into psychological readiness for activity resumption. Consistent with these findings, Ardern et al. reported that psychological readiness and fear of reinjury were significant predictors of returning to preinjury sport levels, despite adequate physical recovery (Ardern et al., 2013). Similarly, Baez et al. found that lower psychological readiness and greater injury-related fear were associated with persistent knee symptoms after ACL reconstruction, highlighting the ongoing influence of psychological factors during recovery (Baez et al., 2023). In line with this perspective, Faleide and Inderhaug emphasized that rehabilitation approaches should address psychological readiness alongside physical criteria when evaluating return-to-sport preparedness (Faleide and Inderhaug, 2023).

The findings also align with emerging evidence emphasizing the neurocognitive and perceptual dimensions of ACL rehabilitation. Narratives frequently reflected altered proprioceptive awareness, feelings of bodily disconnection, and compulsive monitoring of knee-related sensations. Such experiences may reflect disruptions not only in physical function, but also in the individual’s confidence in the predictability and reliability of the injured body (Longo et al., 2023). From a clinical perspective, these findings suggest that postoperative rehabilitation may benefit from incorporating structured reassurance, expectation management, and psychologically informed communication strategies alongside traditional biomechanical recovery protocols.

### 4.2 Fear beyond reinjury: identity, independence, and financial vulnerability

A second major theme involved the broader psychosocial consequences of ACL injury. Across narratives, fears frequently extended beyond reinjury itself and centered on disruptions in occupational identity, financial stability, activity participation, and personal independence. These findings converge with, and add first-person texture to, prior research documenting the social and contextual determinants of psychological recovery after ACL injury (Du et al., 2022; Nedder et al., 2025).

Several narratives reflected profound anxiety regarding career interruption, inability to maintain employment, or loss of future opportunities. For some individuals, the injury appeared to threaten not only physical functioning but also core aspects of self-identity and autonomy. This was particularly evident in narratives describing occupations or lifestyles heavily dependent on physical performance. Such findings reinforce the importance of understanding ACL rehabilitation within a broader life-context framework rather than viewing recovery solely through surgical or performance-based outcomes (Troyer et al., 2025; Gamble et al., 2024).

The findings additionally suggest that socioeconomic and occupational pressures may substantially shape rehabilitation experiences and return-to-sport decision-making processes. Participants described delaying surgical intervention, concerns regarding prolonged absence from work, and consideration of premature return to activity because of financial responsibilities. Although the present study cannot establish causal relationships, these narratives indicate that external socioeconomic stressors may intensify psychological distress and influence rehabilitation behaviors. This interpretation is consistent with previous literature demonstrating that socioeconomic disadvantage can negatively affect ACL injury management and recovery outcomes. For instance, Patel et al. reported that lower socioeconomic status was associated with poorer postoperative outcomes following ACL reconstruction (Patel et al., 2019), while Stafford et al. identified barriers such as treatment costs, delayed access to care, and limited insurance coverage as important contributors to disparities in ACL management (Stafford et al., 2022). Furthermore, emerging rehabilitation literature suggests that insufficient financial and social support may interfere with adherence to rehabilitation processes and long-term recovery trajectories (Liu et al., 2025; Walker et al., 2020). Together, these findings support the importance of integrating occupational and socioeconomic considerations into patient-centered rehabilitation planning in order to promote more sustainable physical and psychological recovery.

### 4.3 Uncertainty, digital reassurance seeking, and the need for clinical guidance

A third major finding concerned the prominent role of uncertainty and reassurance-seeking throughout the rehabilitation process. Participants frequently described searching online forums, comparing rehabilitation timelines with those of other patients, repeatedly monitoring bodily sensations, and seeking reassurance regarding reinjury risk and return-to-sport readiness. Similar experiences have been reported in previous qualitative ACL research. Piussi et al. demonstrated that individuals recovering from ACL injury commonly experience uncertainty regarding knee function, future sports participation, and whether their recovery progress is “normal,” particularly when rehabilitation trajectories differ from expected timelines (Piussi et al., 2022). Previous research has additionally shown that fear of reinjury and uncertainty about bodily safety may contribute to increased symptom monitoring and reduced confidence during return-to-sport decision-making. Ardern et al. identified psychological factors such as fear, lack of confidence, and uncertainty as major barriers to successful return to sport after ACL reconstruction (Ardern et al., 2013). Together, these findings suggest that uncertainty may not simply reflect insufficient information, but also an ongoing difficulty in re-establishing trust in the injured body. Consequently, rehabilitation approaches incorporating consistent clinician communication, expectation management, and psychologically informed support strategies may help reduce anxiety and improve perceived recovery security.

The anonymous and community-driven structure of Reddit appeared to function simultaneously as a source of emotional support and a mechanism for anxiety amplification. While some users sought reassurance and normalization of symptoms through peer interaction, others reported increased distress following exposure to “horror stories,” negative recovery narratives, or conflicting rehabilitation advice shared online. This dual role is consistent with previous research demonstrating that online health communities can both enhance perceived social support and contribute to heightened health anxiety and uncertainty through exposure to emotionally charged or contradictory information (Ziebland and Wyke, 2012; Sharma et al., 2006). These findings further reflect the growing influence of digital health ecosystems in shaping patient perceptions of recovery, risk, and self-management practices.

Narratives reflecting insufficient clinical guidance frequently described feelings of abandonment, confusion, and distrust regarding rehabilitation progression. Kuenze et al., in their qualitative study examining adolescent patient, parent, and clinician perceptions following ACL reconstruction, highlighted that inconsistent information sharing and inadequate interpersonal communication contributed substantially to uncertainty throughout rehabilitation (Kuenze et al., 2022). Similarly, Welling et al. reported that negative rehabilitation experiences were commonly associated with a lack of attention, insufficient professional guidance, absence of goal setting, and limited individualized support from physiotherapists (Welling et al., 2022). Together, these findings suggest that reassurance during ACL rehabilitation extends beyond the provision of technical explanations and also involves restoring predictability, confidence, and a sense of safety throughout recovery. Consequently, clinician guidance may represent a psychologically important component (i.e., ‘...clinician guidance may represent a psychologically important component of postoperative care...’) of postoperative care rather than merely informational interventions (Van Haren et al., 2026; Welling et al., 2022).

### 4.4 Clinical implications

Taken together, the findings of this study support a biopsychosocial understanding of ACL rehabilitation. The narratives analyzed suggest that kinesiophobia following ACL injury may involve not only fear of reinjury, but also broader disruptions in bodily trust, self-identity, emotional security, and future planning. Consequently, rehabilitation strategies focusing exclusively on mechanical stability and physical performance may insufficiently address the psychological dimensions of recovery.

Clinicians may therefore benefit from incorporating psychologically informed approaches into ACL rehabilitation, including expectation management, reassurance-based communication, gradual exposure to feared movements, and acknowledgment of occupational or social stressors influencing recovery experiences. Such approaches may help improve psychological readiness and patient confidence during return-to-activity processes. Concretely, this study’s findings point to a specific, actionable gap in current orthopedic practice: standard postoperative rehabilitation protocols typically specify criteria and timelines for biomechanical and functional milestones (strength symmetry, hop-test performance, range of motion) but rarely include a corresponding, systematic mental health checkpoint. Embedding a brief, routine screening for fear-avoidance beliefs at key rehabilitation milestones, with a defined referral pathway to a psychologist or psychiatrist when indicated, would translate the biopsychosocial understanding supported by this study into a concrete addition to existing orthopedic care pathways, rather than a general appeal for more holistic care.

### 4.5 Limitations

Several limitations should be considered when interpreting the findings of this study. First, clinical diagnoses, surgical details, and rehabilitation status could not be verified against medical records. The unstructured, self-reported format that gives access to spontaneous emotional expression is the same format that precludes verification. This trade-off is inherent to netnographic research rather than a contradiction of its rationale. Second, Reddit users may not represent the broader population of individuals with ACL injury, particularly older adults or individuals with limited access to online communities. Third, only English-language written narratives were included, which may limit cultural generalizability. Additionally, the study focused exclusively on original posts and did not analyze user comments or interpersonal interactions within discussion threads.

Fourth, the search strategy itself constitutes an important methodological limitation. Narratives were identified using a single combined keyword query (“ACL” AND “kinesiophobia”) entered on one occasion (April 25, 2026), which likely excluded conceptually relevant posts describing fear-related experiences through alternative terminology (e.g., “fear of movement,” “scared to reinjure,” “afraid to run”) without explicitly using the term “kinesiophobia.” In addition, Reddit’s search and ranking algorithm is proprietary, dynamically generated, and not fully reproducible; repeating the same query at a different time point, or from a different account, may yield a different set or ordering of results. Because sampling relied on relevance-based ranking rather than an exhaustive or algorithmically transparent retrieval process, the dataset should be regarded as a purposive, illustrative sample of relevant narratives rather than a representative or complete census of ACL-related kinesiophobia discourse on Reddit. Finally, the search was restricted to a single platform; relevant experiences shared on other forums, Facebook support groups, or other social media platforms were not captured, which may have introduced platform-specific selection bias.

Despite these limitations, the use of naturally occurring online narratives provided access to spontaneous and emotionally detailed experiences that may not emerge as openly within traditional clinical interviews. The findings therefore offer valuable insight into the lived psychological experiences associated with ACL rehabilitation and kinesiophobia within contemporary digital health environments.

## 5 Conclusion

We set out to examine how individuals recovering from ACL injury describe and make sense of movement-related fear. Drawing on 50 first-person Reddit narratives, the analysis identified three interrelated dimensions of this experience: a progressive loss of trust in the injured body despite objective recovery; fear-driven disruption of occupational and functional identity and financial security; and persistent uncertainty sustained by inconsistent guidance and unfavorable social comparison. Integrating a structured mental health component, informed by these dimensions, into existing orthopedic rehabilitation pathways may improve both emotional recovery and return-to-activity readiness following ACL injury.

## Data Availability

The data used in this study were obtained from publicly accessible Reddit posts identified through Reddit’s internal search engine using the query “ACL kinesiophobia” (https://www.reddit.com/search/?q=ACL+Kinesiophobia). Because Reddit content is continuously updated and posts may be edited or deleted over time, the exact dataset analyzed in this study is not permanently reproducible through the search link alone. Due to ethical considerations and Reddit’s content policies, the full dataset is not publicly shared but is available from the corresponding author upon reasonable request.
