# Supplementary material for: Kinesiophobia following ACL reconstruction: a qualitative netnographic study of lived experiences shared on Reddit
Source: Front Psychol. 2026 Oct 14;17:1926382. doi: 10.3389/fpsyg.2026.1926382 (PMC13713374; doi:10.3389/fpsyg.2026.1926382)
Supplement: Supplementary file 1 [file Data_Sheet_1.pdf]

SRQR (Standards for Reporting Qualitative Research) Checklist

Completed according to O'Brien BC, Harris IB, Beckman TJ, Reed DA, Cook DA. Standards for reporting qualitative research: a synthesis of recommendations. Acad Med. 2014;89:1245-1251. Manuscript title: "Kinesiophobia Following ACL Reconstruction: A Qualitative Netnographic Study of Lived Experiences Shared on Reddit."

No.	Topic	Item	Reported	Location in Manuscript
**TITLE AND ABSTRACT**				
1	Title	Concise description of the nature and topic of the study; identifying the study as qualitative or indicating the approach/data collection method is recommended.	Yes	Title page — "...A Qualitative Netnographic Study..."
2	Abstract	Summary of key elements (background, purpose, methods, results, conclusions) using the target journal's abstract format.	Yes	Abstract
**INTRODUCTION**				
3	Problem formulation	Description and significance of the problem studied; review of relevant theory and empirical work; problem statement.	Yes	Section 1, Introduction
4	Purpose / research question	Purpose of the study and specific objectives or questions.	Yes	Section 1, final paragraph
**METHODS**				
5	Qualitative approach and research paradigm	Qualitative approach (e.g., netnography) and guiding theoretical framework; rationale.	Yes	Section 2.1, Study Design
6	Researcher characteristics and reflexivity	Researchers' characteristics that may influence the research; potential interaction between researcher characteristics and the study.	Yes	Section 2.5, final paragraph (reflexivity)
7	Context	Setting/site and salient contextual factors; rationale.	Yes	Section 2.2, Data Source and Search Strategy
8	Sampling strategy	How and why participants/documents/events were selected; criteria for ending sampling (e.g., saturation); rationale.	Yes	Section 2.3, Sampling and Eligibility Criteria
9	Ethical issues pertaining to human subjects	Documentation of ethics board approval or waiver, and informed consent if applicable.	Partial	Section 2.6, Ethical Considerations — waiver rationale could be further specified with the exact institutional policy/regulation cited
10	Data collection	Types of data collected;	Yes	Sections 2.2–2.3

	methods	procedures, including start/stop dates, iterative process, and any modification of procedures.		
11	Data collection instruments and technologies	Description of instruments/devices used for data collection.	Yes	Section 2.2 — Reddit's internal search engine
12	Units of study	Number and relevant characteristics of participants/documents/events included.	Yes	Table 1; Section 3, first paragraph
13	Data processing	Transcription, data entry, management/security, verification of integrity, coding, and de-identification of excerpts.	Yes	Sections 2.4 and 2.6; Supplementary Table S1
14	Data analysis	Process by which themes were identified and developed, including researchers involved; paradigm/approach; rationale.	Yes	Section 2.5, Data Analysis
15	Techniques to enhance trustworthiness	Techniques used (e.g., triangulation, audit trail, member checking) and rationale.	Yes	Section 2.5 — investigator triangulation and interdisciplinary interpretation
**RESULTS / FINDINGS**				
16	Synthesis and interpretation	Main findings (themes, categories) with links to data/quotations demonstrating findings-interpretation connections.	Yes	Section 3.1–3.3; Table 2
17	Links to empirical data	Evidence (quotes, excerpts) to substantiate analytic findings.	Yes	Section 3.1–3.3 (paraphrased illustrative excerpts; see Section 2.6 for rationale)
**DISCUSSION**				
18	Integration with prior work, implications, transferability, contribution(s)	Summary of findings; connection to prior scholarship; scope of application; unique contribution.	Yes	Sections 4.1–4.4
19	Limitations	Trustworthiness and limitations of findings.	Yes	Section 4.5, Limitations
**OTHER**				
20	Conflicts of interest	Sources of funding and other support; management of conflicts of interest.	Yes	Declarations — Conflict of Interest
21	Funding	Sources of funding and other support.	Yes	Declarations — Funding

Note: Item 9 is marked "Partial." Reviewers may request that the ethics waiver statement in Section 2.6 name the specific institutional policy or national regulation under which formal IRB approval was not required.
