# Supplementary material for: Kinesiophobia following ACL reconstruction: a qualitative netnographic study of lived experiences shared on Reddit
Source: Front Psychol. 2026 Oct 14;17:1926382. doi: 10.3389/fpsyg.2026.1926382 (PMC13713374; doi:10.3389/fpsyg.2026.1926382)
Supplement: Supplementary file 2 [file Supplementary_file_1.pdf]

Supplementary Table S1. Codebook summarizing the final coding framework, code definitions, and illustrative (paraphrased) excerpts

This codebook summarizes the coding structure used during reflexive thematic analysis of the 50 included Reddit narratives. Coding was conducted manually using structured Word/Excel tables by the six-member research team (three orthopedic surgeons, three psychiatrists). Illustrative excerpts shown below correspond to the paraphrased excerpts reported in the main manuscript; original verbatim narratives are retained by the authors and are not publicly shared, in order to protect user anonymity.

Code	Definition / Inclusion Criterion	Illustrative Excerpt (paraphrased)	Narrative
**Theme 1: Loss of Trust in the Injured Body**			
Hypervigilance toward bodily sensations	Ordinary postoperative sensations (clicking, cracking, stiffness) are closely monitored and interpreted as warning signs.	"Any small pop or click in my knee makes my stomach lurch, and my first thought is always that I've retorn the graft."	N7
Catastrophic interpretation of normal recovery	Expected recovery-related experiences are reinterpreted as evidence of structural failure or permanent damage.	"I lost hours last night searching online to find out whether a graft can tear without any swelling."	N30
Altered proprioceptive awareness / bodily disconnection	Perceived loss of normal sensory-motor connection to the injured limb.	"My quad feels totally lifeless, as if the signal between my brain and that muscle has been switched off."	N6
Discrepancy between objective recovery and subjective safety	Continued protective or cautious behavior despite satisfactory strength/functional testing.	"My strength tests show my operated leg matching the healthy one, but mentally I still handle it as if it might shatter."	N23
Persistent fear-avoidance despite medical clearance	Freeze or avoidance response to movement-related triggers even after return-to-sport clearance.	"During a basketball game, another player's foot came close to mine and I froze on the spot."	N4
**Theme 2: Fear-Driven Identity and Future Disruption**			
Threat to occupational/athletic identity	Injury experienced as a disruption to core professional or athletic self-concept.	"This single injury feels like it has wiped out my career, my sense of who I am, and my financial security all at once."	N16
Financial anxiety / economic vulnerability	Concerns about income loss, medical costs, or ability to meet financial obligations.	"What frightens me most about needing surgery is not knowing how I would keep up with my mortgage payments."	N50
Fear of dependency / loss of autonomy	Anticipatory distress about needing physical assistance	"Honestly, the idea of relying on someone else scares me	N46

	from others during recovery.	more than I expected."	
Behavioral avoidance due to recurrence fear	Long-term lifestyle or activity modification following one or more reinjuries.	"Since my second tear, I've become extremely careful and avoid anything that feels remotely risky."	N48
Emotional exhaustion / hopelessness with recurrent injury	Expressions of dread or fatigue associated with repeating the rehabilitation process.	"I still can't wrap my head around having to go through this whole ordeal again from square one."	N32
**Theme 3: Uncertainty, Information Seeking, and Need for Clinical Reassurance**			
Anticipatory / preoperative anxiety	Fear and rumination prior to surgery, often fueled by negative online accounts.	"I keep coming across frightening stories online about permanent kneeling pain and grafts failing."	N2
Perceived inadequate clinical guidance	Reports of insufficient or unclear communication regarding rehabilitation protocol.	"I still haven't received a physical therapy schedule and feel like I have no direction at all."	N37
Social comparison via online platforms	Comparing personal recovery trajectory unfavorably against others' shared timelines online.	"Watching other people online already running again by three months makes me feel like I'm falling behind."	N11
Reassurance-seeking / symptom-checking behavior	Directly soliciting other users' experiences to validate or normalize own symptoms.	"What does knee instability actually feel like for those who have it?"	N42

Note: Illustrative excerpts are paraphrased versions of the original Reddit narratives, consistent with the data-masking approach described in Section 2.6 of the manuscript, in order to reduce the risk of re-identification through reverse text-search. Raw verbatim narratives were used only for internal coding and are retained by the corresponding author; they are not publicly disclosed to protect participant anonymity.
